# The hypoxia sensitive metal transcription factor MTF-1 activates NCX1 brain promoter and participates in remote postconditioning neuroprotection in stroke

**DOI:** 10.1038/s41419-021-03705-9

**Published:** 2021-04-30

**Authors:** Valeria Valsecchi, Giusy Laudati, Ornella Cuomo, Rossana Sirabella, Annalisa Del Prete, Lucio Annunziato, Giuseppe Pignataro

**Affiliations:** 1 Division of Pharmacology, Department of Neuroscience, Reproductive and Dentistry Sciences, School of Medicine, Federico II University of Naples, via Pansini 5, 80131 Naples, Italy; 2 IRCCS SDN, via Gianturco 113, 80143 Naples, Italy

**Keywords:** Cellular neuroscience, Preclinical research

## Abstract

Remote limb ischemic postconditioning (RLIP) is an experimental strategy in which short femoral artery ischemia reduces brain damage induced by a previous harmful ischemic insult. Ionic homeostasis maintenance in the CNS seems to play a relevant role in mediating RLIP neuroprotection and among the effectors, the sodium-calcium exchanger 1 (NCX1) may give an important contribution, being expressed in all CNS cells involved in brain ischemic pathophysiology. The aim of this work was to investigate whether the metal responsive transcription factor 1 (MTF-1), an important hypoxia sensitive transcription factor, may (i) interact and regulate NCX1, and (ii) play a role in the neuroprotective effect mediated by RLIP through NCX1 activation. Here we demonstrated that in brain ischemia induced by transient middle cerebral occlusion (tMCAO), MTF-1 is triggered by a subsequent temporary femoral artery occlusion (FAO) and represents a mediator of endogenous neuroprotection. More importantly, we showed that MTF-1 translocates to the nucleus where it binds the metal responsive element (MRE) located at −23/−17 bp of *Ncx1* brain promoter thus activating its transcription and inducing an upregulation of NCX1 that has been demonstrated to be neuroprotective. Furthermore, RLIP restored MTF-1 and NCX1 protein levels in the ischemic rat brain cortex and the silencing of MTF-1 prevented the increase of NCX1 observed in RLIP protected rats, thus demonstrating a direct regulation of NCX1 by MTF-1 in the ischemic cortex of rat exposed to tMCAO followed by FAO. Moreover, silencing of MTF-1 significantly reduced the neuroprotective effect elicited by RLIP as demonstrated by the enlargement of brain infarct volume observed in rats subjected to RLIP and treated with MTF-1 silencing. Overall, MTF-dependent activation of NCX1 and their upregulation elicited by RLIP, besides unraveling a new molecular pathway of neuroprotection during brain ischemia, might represent an additional mechanism to intervene in stroke pathophysiology.

## Introduction

In the last decade, remote limb ischemic postconditioning (RLIP) emerged as a potent neuroprotective strategy. In fact, it has been reported that sub-lethal ischemia applied to the femoral artery is able to effectively reduce brain damage from a previous harmful ischemic insult^1^. Several events have been involved in the neuroprotection exerted by RLIP such as brain edema attenuation, reduced inflammation, blood–brain barrier (BBB) preservation^2^. In most of these protective mechanisms, ionic homeostasis maintenance seems to play a relevant role. Among the effectors of ionic homeostasis maintenance, the sodium-calcium exchanger 1 (NCX1) may give an important contribution being expressed in all CNS cells, including neurons and glial cells^3,4^, where it can operate in the forward mode-coupling the extrusion of Ca^2+^ and the entrance of Na^+^ ions or in the reverse mode. Considering that in the ischemic core a dramatic reduction of ATP occurs, it is conceivable to hypothesize that the absence of ATP impairs all ATP-dependent transporters, i.e., Na^+^/K^+^ATPase, thus forcing NCX to work in the reverse mode. The situation should be different in the penumbra region where ATP is still present. For these peculiar functions, NCX1 may counteract the ionic homeostasis dysregulation, that occurs during an ischemic insult, extruding Na^+^ ions from the cell and promoting Ca^2+^ refilling of the ER^5^, thus preserving the intracellular Ca^2+^ and Na^+^ concentrations within physiological levels in the brain^6^. Indeed, brain damage worsens in ischemic animals in which NCX1 is knocked out by antisense oligodeoxynucleotides^7^ or in which NCX1 was genetically ablated and ameliorates when NCX1 is overexpressed^7^.

In the last few years, in order to develop drugs modulating the expression and the activity of this exchanger, there has been a growing interest in identifying and characterizing new transcriptional factors and epigenetic modulators of the *Slc8a1* gene that codes for NCX1 protein. Notably, among the stroke-induced transcriptional regulators of the NCX1 gene, the hypoxia-inducible factor-1 (HIF-1)^8^, the specific protein-1 (Sp1), and the RE1-silencing transcription factor (REST) have been identified as important modulators. Interestingly, while the NCX1 activators HIF-1 and Sp1 take part in neuroprotection, the NCX1 repressor factor REST worsens stroke-induced brain damage^9,10^.

On the other hand, we considered that another important hypoxia-sensitive transcription factor is the metal responsive transcription factor 1 (MTF-1). In fact, MTF-1 is a protein evolutionarily conserved from insects to humans and its DNA binding domain, consisting of six zinc fingers^11^, is able to interact with specific promoter regions of target genes known as metal response elements (MREs)^12,13^. Interestingly, MTF-1 plays a pivotal role in counteracting the effects of heavy metal overload by inducing the expression of genes coding for metallothioneins (MTs)^14,15^.

In the light of the properties of MTF-1, we investigated whether: (i) *Slc8a1* gene might be a target of the transcriptional factor MTF-1 and (ii) MTF-1 might play a role in the neuroprotective effect mediated by RLIP through NCX1 activation.

## Results

### The metal transcription factor MTF-1 participates in the activation of the brain *ncx1* promoter

**Fig. 1:**
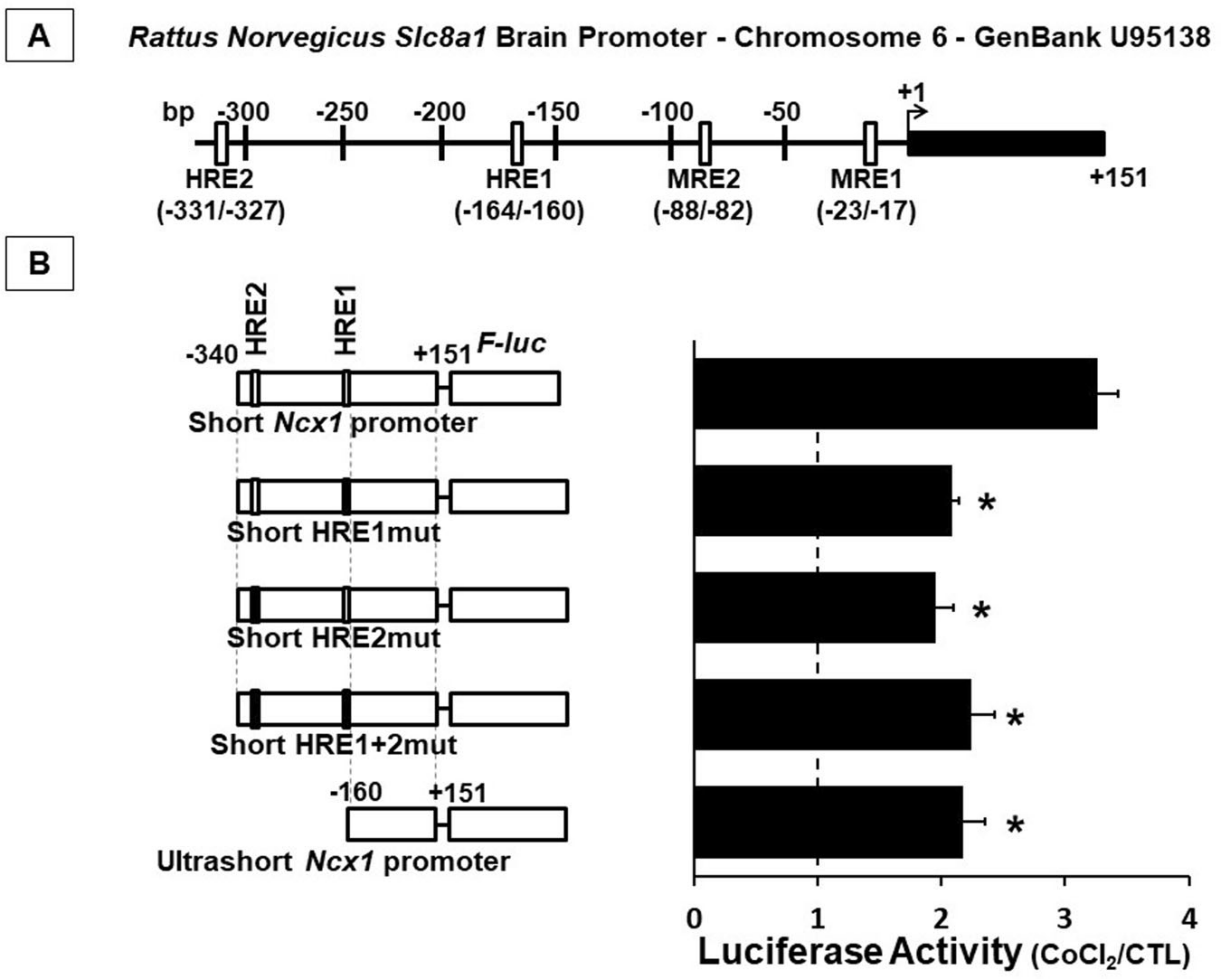
HIF-1 activated the Na^+^/Ca^2+^ exchanger brain *Slc8a1* promoter. **A** Diagram showing the structure of the *Rattus Norvegicus* Na^+^/Ca^2+^ exchanger brain *Slc8a1* promoter. HIF-1 and MTF-1 putative responsive elements (HRE-MRE) are indicated by open rectangles. The transcription start site is indicated as +1. The mRNA sequence is indicated by a black rectangle. **B** Luciferase assay, expressed CoCl_2_/CTL ratio, of SH-SY5Y cells transfected with short ncx1 (−340/+151) promoter or short HRE1mut or short HRE2mut or short HRE1-2mut, or ultrashort ncx1 promoter (−160/+151) incubated with 100 μM of CoCl_2_ for 24 h. *p < 0.05 vs. short ncx1 (−340/+151) promoter by one-way ANOVA followed by Bonferroni test. Each column represents the mean ± SEM of 3/10 independent experimental sessions, each one run in duplicate.

Computational analysis (TRANSFAC version 3.2) of the short *Ncx1* brain promoter sequence (−330/+151 of GenBank sequence accession number U95138) revealed the presence of two putative metal responsive elements for MTF-1, MRE1 at −23/−17 bp from the transcriptional start site and MRE2 at −88/−82 bp, in close proximity to the *consensus* sites for the hypoxia-induced NCX1 activators HIF-1 and Sp1^9^. In order to investigate MTF-1 contribution to *Ncx1* transcriptional activation, we used cobalt chloride as an activator of MTF-1^16^. Preliminarily, we intended to exclude that the cobalt-induced activation of MRE1 and MRE2 sites by MTF-1 was dependent from HRE1 and HRE2 activation by HIF-1, as previously reported^9^. To this aim, we mutated or deleted the HREs and evaluated luciferase activity following CoCl_2_ exposure. In particular, we carried out luciferase assay by mutating individually or together the two *consensus* sequences for HIF-1: the HRE1 located at −164/−160 bp and the HRE2 located at −331/−327 bp (Fig. 1A). A shorter 160-bp fragment of brain *Ncx1* promoter, lacking the two HREs located upstream, was also generated. We confirmed that CoCl_2_ increased luciferase activity of the short *Ncx1* promoter by 3.3 fold^9^. Interestingly, single mutations either of HRE1 or HRE2 reduced luciferase increase following CoCl_2_ exposure approximately by 40% compared to wild type short *Ncx1* promoter (Fig. 1B). However, the double mutant did not further reduce luciferase response following CoCl_2_ compared to the short HRE1mut or to the short HRE2mut, and more importantly, luciferase activity of the short HRE1+2mut vector was 2.2-fold higher than the respective control (Fig. 1B), thus suggesting the presence of other transcription factors able to activate *Ncx1* brain promoter following cobalt stimulation. Luciferase response following CoCl_2_ of the ultrashort *Ncx1* promoter was comparable to that of the short Ncx1 promoter, therefore, we used this construct for further experiments aimed to identify new transcription factor modulating *Ncx1* independent from HREs and HIF-1 contribution.

**Fig. 2:**
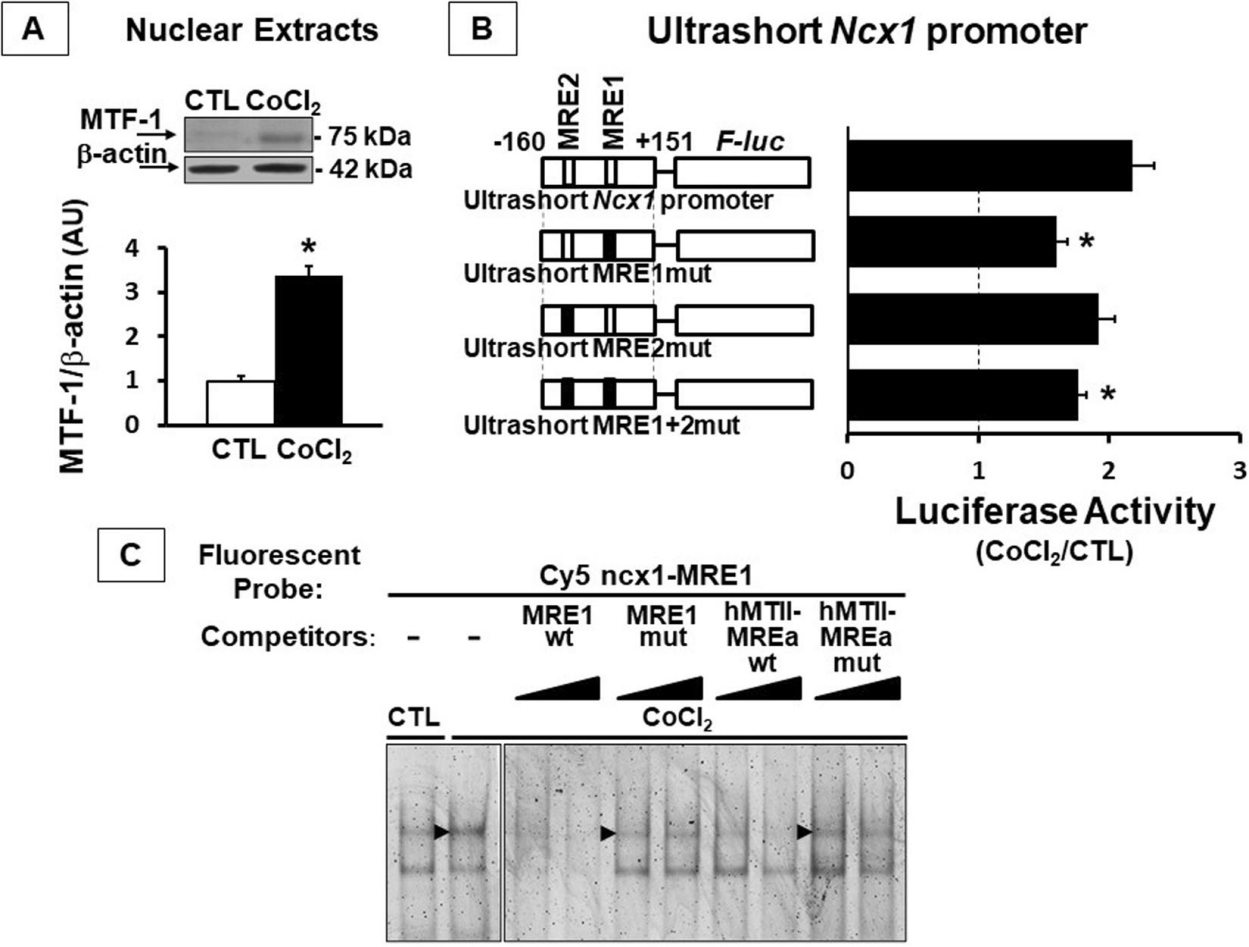
MTF-1 activated the Na^+^/Ca^2+^ exchanger brain *Slc8a1* promoter. **A** Representative Western blot of MTF-1 in a nuclear extract from SH-SY5Y cells treated with CoCl_2_. **B** Luciferase assay, expressed CoCl_2_/CTL ratio, of SH-SY5Y cells transfected with ultrashort ncx1 (−160/+151) promoter or ultrashort MRE1mut or ultrashort MRE2mut or ultrashort MRE1+2mut, incubated with 100 μM of CoCl_2_ for 24 h. **C** EMSA on nuclear extracts from SH-SY5Y cells untreated (CTL; lane 1) or treated with 100 μM of CoCl_2_ for 24 h incubated with the Cy5-tagged ncx1-MRE1 probe (CoCl_2_; lanes 2/10). Competition experiments were performed with increasing quantities (50× and 100×) of: the unlabeled wild-type MRE1 (ncx1-MRE1 wt; lanes 3–4); the mutant MRE1 (ncx1-MRE1 mut; lanes 5–6); the wild type MRE of the *Homo Sapiens* metallothionein II gene (hMTII-MREa wt; lanes 7–8); and the mutant metallothionein II MRE (hMTII-MREa mut; B, lane 4) oligonucletides. *p < 0.05 vs. ultrashort ncx1 (−160/+151) promoter by one-way ANOVA followed by Bonferroni test. Each column represents the mean ± SEM of 4/9 independent experimental sessions, each one run in duplicate.

Interestingly, by WB analysis on nuclear extracts from SH-SY5Y, we verified that MTF-1 increased after CoCl_2_ exposure by 3.4 fold compared to control cells (Fig. 2A). To assess whether the two putative MRE *consensus* sequences might contribute to *Ncx1* transcriptional activation following CoCl_2_, we mutated the MREs individually or together (Fig. 2B). Interestingly, luciferase assay showed reduced activation only when the MRE1 site was mutated in the ultrashort MRE1mut and ultrashort MRE1+2mut vectors. In fact, the reduction in luciferase assay was 30% and 20%, respectively, compared to ultrashort *Ncx1* promoter (−160/+151). Cells transfected with ultrashort MRE2mut did not show a significant difference in luciferase increase compared to the wild-type vector (Fig. 2B). Notably, cells transfected with the ultrashort MRE1mut vector still showed a 1.6-fold increase in luciferase activity following cobalt exposure compared to control cells (Fig. 2B).

In order to further confirm MTF-1 binding to *Ncx1* promoter, we performed EMSA on MRE1 sequence placed at −23/−17 bp within the brain *Ncx1* promoter. Nuclear extracts from CoCl_2_-exposed cells were incubated with a 24-bp Cy5-fluorescent probe (ncx1-MRE1) containing the MRE1 site. Protein binding to the fluorescent probe in cobalt-treated nuclear extracts produced a very intense shifted band compared to untreated cells (Fig. 2C, lanes 1–2). To verify the specific binding of MTF-1 protein to the fluorescent probe, competition experiments were performed. Thus, by using increasing quantities of an ncx1-MRE1 oligonucleotide, the fluorescent ncx1-MRE1 complex was completely displaced (Fig. 2C, lanes 3–4). This competition did not occur in the presence of an unlabeled mutated probe (Fig. 2C, lanes 5–6). When we used as a competitor the hMTII-MREa oligonucleotide, which contained the MRE for human metallothionein gene, a known target sequence of MTF-1^17^, the complex formation was completely prevented (Fig. 2C, lanes 7–8). Finally, the competition carried out by the wild-type hMTII-MREa oligonucleotide did not occur when nuclear extracts from CoCl_2_-exposed cells were incubated with a mutated hMTII-MREa oligonucleotide (Fig. 2C, lanes 9–10).

However, co-immunoprecipitation experiments between HIF-1 and MTF-1 in SH-SY5Y cells exposed to CoCl_2_ did not show any interaction between these proteins (data not shown).

### The transcription factor Sp1 activates the ultrashort *Ncx1* promoter after exposure to CoCl_2_

**Fig. 3:**
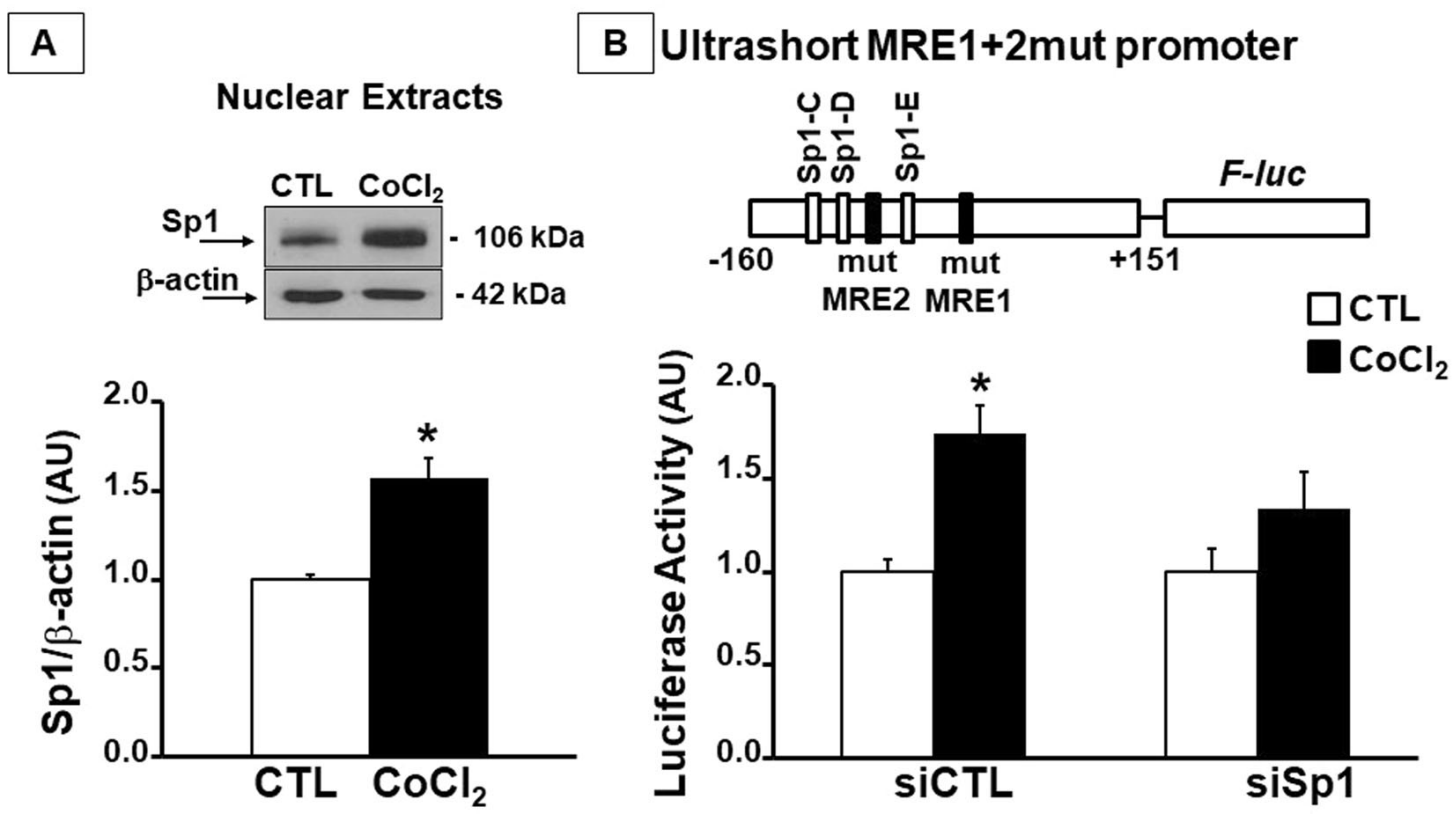
Sp1 was able to modulate the Na^+^/Ca^2+^ exchanger brain *Slc8a1* promoter following CoCl_2_ treatment. **A** Representative Western blot of Sp1 in a nuclear extract from SH-SY5Y cells treated with CoCl_2_. **B** Luciferase assay of SH-SY5Y cells co-transfected with the ultrashort MRE1+2mut promoter and non-targeting siRNA (siCTL) or siRNA for Sp1 (siSp1), untreated (CTL) or treated with 100 μM of CoCl_2_ for 24 h. *p < 0.05 vs. CTL by *t*-test for panel **A**. *p < 0.05 vs. CTL by one-way ANOVA followed by Bonferroni test for panel **B**. Each column represents the mean ± SEM of 6 independent experimental sessions, each one run in duplicate.

Notably, cells transfected with the ultrashort MRE1+2mut vector still showed a slight but significant increase in luciferase activity following cobalt exposure compared to control cells (Fig. 2B), suggesting the presence of other transcription factors activated by cobalt exposure. Therefore, we considered also the Sp1 *consensus* sites located at −129/−121, −111/−104, and −67/−58 bp from the transcriptional start site^10^. Surprisingly, cobalt chloride slightly increased Sp1 levels by 1.5-fold in the nuclear compartment of SH-SY5Y cells compared to control ones (Fig. 3A). Luciferase assay experiments were carried out in SH-SY5Y cells co-transfected with the ultrashort MRE1+2mut vector and a siRNA able to specifically knockdown Sp1 (siSp1). The efficiency of siRNA was verified by WB assay (Fig. S1A). Indeed, SH-SY5Y cells transfected with siSp1 did not display any increase in Sp1 level after CoCl_2_ exposure, whereas a non-targeting siRNA (siCTL) was not able to counteract cobalt-induced Sp1 upregulation (Fig. 3B).

Luciferase activity of CoCl_2_-exposed cells co-transfected with the ultrashort MRE1+2mut vector and siSp1 did not differ from control cells (Fig. 3B). On the other hand, the siCTL was not able to prevent luciferase increase in CoCl_2_-exposed cells transfected with the ultrashort MRE1+2mut plasmid (Fig. 3B).

### RLIP prevents downregulation of MTF-1 and NCX1 expression induced by tMCAO in the ischemic cortex

**Fig. 4:**
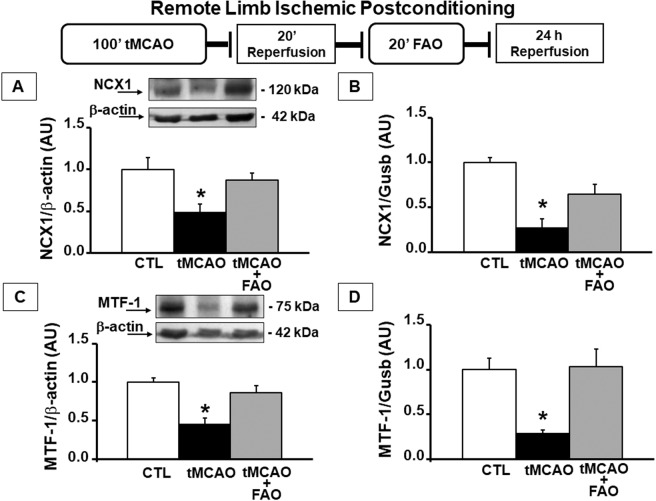
Remote limb ischemic postconditioning was able to revert the downregulation of NCX1 and MTF-1 induced by the ischemic insult. **A** Diagram showing the experimental model of remote limb ischemic postconditioning (RLIP) used. In particular, a 20’ occlusion of the femoral artery (FAO) followed a 100’ of transient middle cerebral artery occlusion (tMCAO), spaced by 20’ of reperfusion. **A**–**C** Real-time PCR for the evaluation of *Ncx1* (**A**) and *Mtf-1* (**C**) expression levels in the cortex of rat: (i) sham-operated (CTL); (ii) exposed to tMCAO (tMCAO); and (iii) tMCAO+FAO. **B**–**D** Representative Western blot of NCX1 (**B**) and MTF-1 (**D**) proteins in the cortex of rat: (i) sham-operated (CTL); (ii) exposed to tMCAO (tMCAO); and (iii) exposed to tMCAO+FAO (tMCAO+FAO). *p < 0.05 vs. CTL by one-way ANOVA followed by Bonferroni test. Each column represents the mean ± SEM (*n* = 3/6).

In order to investigate NCX1 and MTF-1 involvement in RLIP, their expression was evaluated in the rat cortex of ischemic and post-conditioned rats. Interestingly, a strong reduction of NCX1 protein expression of approximately 50% was observed in the ipsilateral temporoparietal cortex of rats subjected to tMCAO followed by 24 h of reperfusion. This reduction was prevented by 20 min of FAO applied after tMCAO (Fig. 4A). To assess whether the increased protein expression of NCX1 in the brain cortex of rats subjected to tMCAO plus FAO was caused by transcriptional activation of the *Ncx1* gene, experiments of RT-PCR were carried out to measure *Ncx1* mRNA levels. Interestingly, our data showed that *Ncx1* mRNA expression was reduced by almost 70% after ischemia alone, whereas tMCAO plus FAO was able to partially restore *Ncx1* mRNA levels (Fig. 4B).

In light of the in vitro results showing the transcriptional regulation of NCX1 by MTF-1 (Fig. 2), we investigated in vivo whether, in the brain cortex of ischemic and post-conditioned rats, MTF-1 could be involved in the increase of NCX1 expression elicited by tMCAO plus FAO. Interestingly, MTF-1 protein and mRNA were reduced by 60% and 70%, respectively, during tMCAO, whereas this reduction was completely prevented by FAO following tMCAO (Fig. 4C–D).

Finally, when the contribution of the other potentially involved transcription factor Sp1 was evaluated, no changes in its expression were detected in the cortex of rats subjected to tMCAO or to tMCAO plus FAO (Fig. S1B).

### Silencing of MTF-1 prevents NCX1 overexpression and neuroprotection elicited by RLIP

To determine whether MTF-1 was involved in the neuroprotection exerted by RLIP through NCX1 overexpression, a mixture containing 4 different types of siRNA for MTF-1 (siMTF-1) was intracerebroventricularly (icv) administered to rats before being subjected to tMCAO or tMCAO plus FAO. The effectiveness of this mixture of siRNAs in knocking-down MTF-1 expression was evaluated by WB analysis (Fig. S2). In particular, icv-administration of siMTF-1 reduced MTF-1 protein expression in rat brain cortex by 40% compared to animals treated with a siCTL (Fig. S2).

**Fig. 5:**
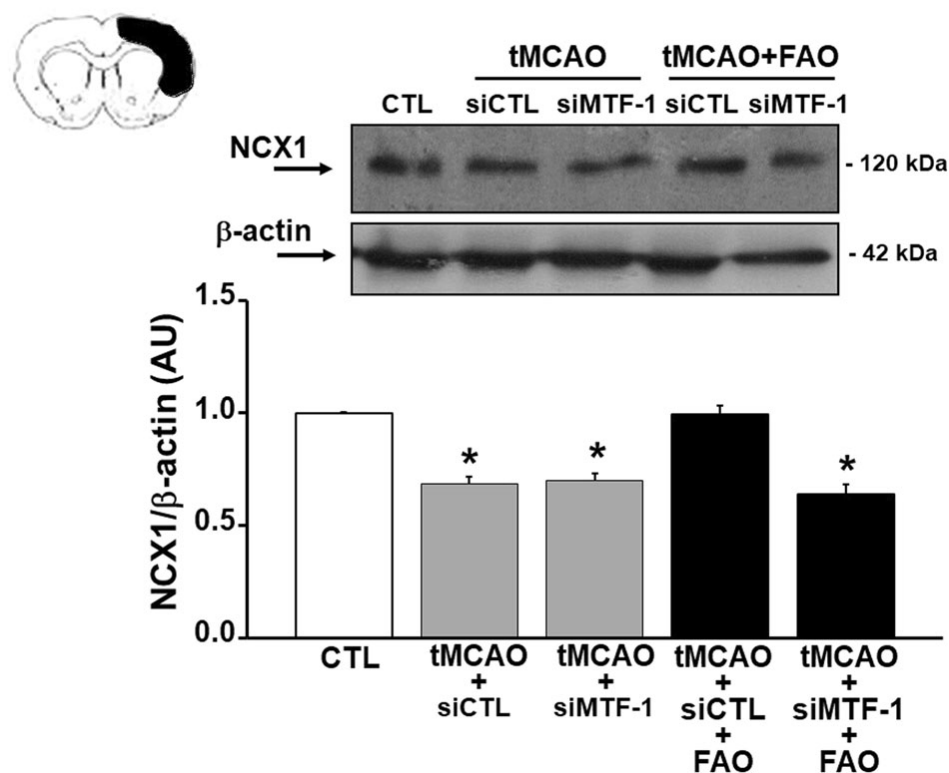
Knocking down MTF-1 by siRNA strategy reverted RLIP-induced upregulation of NCX1. Representative Western blot of NCX1 in the cortex of rat: (i) sham-operated (CTL), (ii) exposed to tMCAO and icv-injected with a non targeting siRNA (tMCAO+siCTL) or a siRNA for MTF-1 (tMCAO+siMTF-1) and; (iii) to tMCAO+FAO icv-injected with a non targeting siRNA (tMCAO+siCTL+FAO) or a siRNA for MTF-1 (tMCAO+siMTF-1+FAO). *p < 0.05 vs. CTL by one-way ANOVA followed by Bonferroni test. Each column represents the mean ± SEM (*n* = 4/5).

**Fig. 6:**
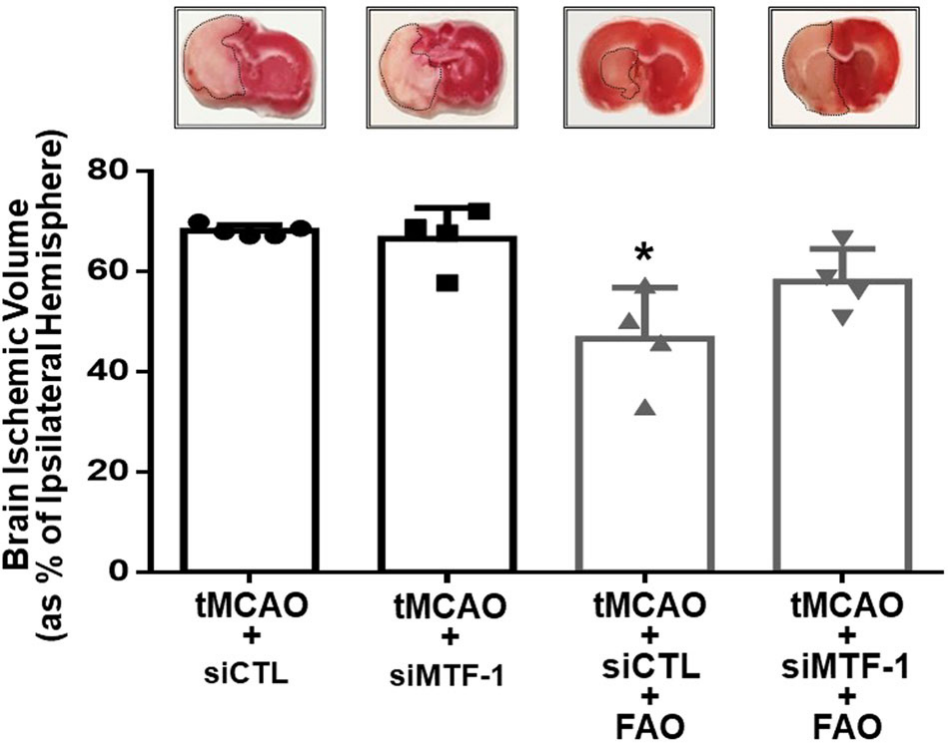
Knocking down MTF-1 by siRNA strategy reverted RLIP-induced neuroprotection. Evaluation of the ischemic damage in rats exposed to: (i) tMCAO and icv-injected with a non targeting siRNA (tMCAO+siCTL) or a siRNA for MTF-1 (tMCAO+siMTF-1) and; (ii) to tMCAO+FAO and icv-injected with a non targeting siRNA (tMCAO+siCTL+FAO) or a siRNA for MTF-1 (tMCAO+siMTF-1+FAO). *p < 0.05 vs. tMCAO+siCTL by one-way ANOVA followed by Bonferroni test. Each column represents the mean ± SEM (*n* = 4/5).

To evaluate the pathophysiological relevance of NCX1 induced by MTF-1 during tMCAO plus FAO, brain NCX1 expression levels and the infarct volume were evaluated in rats subjected to tMCAO or to tMCAO plus FAO previously treated with siMTF or siCTL. Interestingly, NCX1 expression after tMCAO alone was reduced by 30% compared to sham-operated rats (Fig. 5A). Notably, tMCAO plus FAO treatment was able to prevent NCX1 downregulation only in siCTL treated rats (Fig. 5). Indeed, NCX1 upregulation induced by tMCAO plus FAO was completely suppressed by knocking down MTF-1 (Fig. 5). More importantly, the brain infarct volume of rats treated with siCTL and exposed to tMCAO plus FAO was significantly lower than that measured in rats exposed to tMCAO alone, treated either with siCTL or siMTF (Fig. 6). However, the neuroprotective effect exerted by tMCAO plus FAO was partially prevented when MTF-1 was knocked down. Indeed, the infarct volume of tMCAO plus FAO plus siMTF rat brains was not significantly different from that of tMCAO treated animals (Fig. 6).

## Discussion

In the present paper, we demonstrated, for the first time, that^1^: isoform 1 of the sodium–calcium exchanger is a new target of the transcriptional factor MTF-1^2^; the plasma membrane sodium/calcium exchanger 1 may take part in the stroke neuroprotection elicited by remote limb postconditioning; and^3^ MTF-1 mediates NCX1 upregulation occurring in the remote post-conditioned brain, thus taking part to stroke neuroprotection. MTF-1 was firstly described as a pivotal player in counteracting the effects of heavy metal overload by inducing the expression of genes coding for metallothioneins (MTs)^14^, which are small cysteine-rich proteins, able to bind the excess of heavy metal ions (i.e., zinc, copper, chromium, cadmium, mercury)^14^. However, MTF-1 is also involved in the regulation of inflammation, through the activation of pro-inflammatory or anti-inflammatory cytokines^18^, in the regulation of the genes that code for insulin, *insulin1* and *insulin2*, a key player in the regulation of glucose homeostasis^19^. Moreover, this transcription factor is essential for the development processes. Indeed, mice knock-out for MTF-1 dye during the early embryonic state due to the failed liver development^20,21^. Another research study demonstrated that in the brain MTF-1 can be involved in the stimulation of β-synuclein expression, a protein involved in the regulation of neuronal plasticity, and altered MTF-1 levels can contribute to the development of Parkinson’s and Alzheimer’s diseases^22^.

Interestingly, MTF-1 also emerged as an endogenous neuroprotective mechanism against oxidative and hypoxic stress, sensitive to fluctuations in oxygen levels and redox cell status. Indeed, MTF-1 is able to activate the selenoprotein-1 (*Sepw1*) gene, which encodes a glutathione-binding protein provided of antioxidant activity^23^, and MTF-1 expression is increased in preconditioned mouse hippocampal neurons and mediates protection against oxygen and glucose deprivation (OGD)^24^.

In the present paper, we demonstrated that in brain ischemia induced by tMCAO, MTF-1 represents a further mediator of endogenous neuroprotection elicited by remote limb postconditioning, induced by temporary femoral artery occlusion. More importantly, we demonstrated that MTF-1 translocates to the nucleus where it binds the MRE located at −23/−17 bp of *Ncx1* brain promoter, thus activating its transcription and increasing the expression of the ionic exchanger isoform that has been demonstrated to be neuroprotective.

The second MRE sequence identified by computational analysis, located at −88/−82 bp of *Ncx1* brain promoter appears to be not functional under cobalt stimulation.

At variance with MTF-1 that needs the binding to only one *consensus* site, we showed, in this study, that HIF-1 activates *Ncx1* transcription through the binding of two HREs *consensus* sites, located at −331/−327 and −164/−160 bp in respect to the transcriptional starting site^9^. Notably, here we further demonstrated that HIF-1 needs both the HREs to activate *Ncx1* transcription, thus showing that the two HREs are not cooperative. Indeed, site-directed mutagenesis of one of the two HREs abolished HIF-1 contribution to *Ncx1* activation. The requirement of two or more *consensus* sequences for the same transcription factor is a phenomenon already reported for other genes^25^.

Concerning Sp1, in agreement with previous results^10^, we did not observe any changes in its levels after tMCAO, nor after tMCAO followed by FAO. On the other hand, it should be underlined that in this study Sp1 may cause *Ncx1* activation following in vitro exposure to the hypoxia-mimetic agent cobalt chloride. This effect is of particular relevance if one considers that several Sp1 *consensus* sequences are located on the brain *Ncx1* promoter. Indeed, we have previously shown that Sp1 forming a molecular complex with HIF-1 and p300 can activate NCX1 in the ischemic cortex of rat exposed to preconditioning followed by tMCAO^10^.

Another aspect that deserves to be mentioned is that RLIP restored MTF-1 and NCX1 protein levels in the ischemic rat brain cortex and that the silencing of MTF-1 prevented the upregulation of NCX1 observed in RLIP protected rats, demonstrating a direct regulation of NCX1 by MTF-1 in the ischemic cortex of rat exposed to tMCAO followed by FAO. Moreover, silencing of MTF-1 significantly reduced the neuroprotective effect elicited by RLIP as demonstrated by the increased brain infarct volume observed in rats subjected to tMCAO followed by FAO and treated with MTF-1 silencing. In particular, the protocol used for tMCAO induction and subsequent FAO has been previously shown to be the most effective in inducing neuroprotection after stroke^26^. Indeed, a significant reduction in the infarct volume was achieved when animals were subjected to 20’ min of FAO, 20’ after 100’ tMCAO. If the occlusion of the femoral artery lasted less than 10’ or more than 30’ or occurred later than 30’ after tMCAO the neuroprotective effect was not present^26^.

Regarding NCX1 role in brain ischemia, its function has been extensively investigated^27^. It is worthy to mention that NCX1 is activated not only by remote postconditioning, as demonstrated in the present paper, but it is also upregulated when the subthreshold ischemic insult is delivered before tMCAO, i.e., ischemic preconditioning, or after tMCAO, i.e., ischemic postconditioning, where NCX1 silencing reduced the protection^28^, ^29^. In particular, NCX1 and NCX3 are both involved in the neuroprotection elicited by ischemic postconditioning. However, since the MTF-1 *consensus* sequence is present only on the NCX1 brain promoters, we focused on the relationship between NCX1 and MTF-1 in mediating remote postconditioning brain protection.

In addition, the neuroprotective role of NCX1 during stroke has been widely demonstrated by a series of experiments involving pharmacological, genetic, and transcriptional regulation of its expression and activity. In fact, the exposure of ischemic rats to NCX1 and NCX3 oligodeoxynucleotides, silencing or knocking-down the antiporter elicits an increase in the ischemic lesion and a worsening of the rat neurological deficits^30^ whereas, treatment with neurounina-1, a selective activator of NCX1 and NCX2, reduces the infarct volume of adult and neonatal ischemic mice, respectively^31,32^. Analogously, ischemic mice overexpressing NCX1 in the CNS show a reduced infarct volume and amelioration of neurological scores^7^. Finally, administration of the antimiRNA-103, able to counteract the repression exerted by miRNA-103 on NCX1 expression, reduces brain damage and neurological deficits caused by tMCAO^33^. The neuroprotection elicited by NCX activation has been recently confirmed by in vivo multiphoton experiments carried out in mice subjected to permanent MCAO and explained with a reduction of intracellular Na^+^, whose increase seems to be due to NCX activation in the reverse mode. In fact, reverse NCX activity mediates the export of Na^+^ and thereby significantly reduces Na^+^ loads evoked by brain ischemia. Na^+^ influx represents an immediate, major metabolic challenge to both neurons and astrocytes, resulting in the consumption of ATP by the Na^+^/K^+^-ATPase and promoting an ischemia-related decrease in cellular ATP^34^.

Overall, MTF-dependent activation of NCX1 and their upregulation elicited by tMCAO followed by FAO, besides unraveling a new molecular mechanism of neuroprotection during brain ischemia, might pave the way for additional strategies of interventions in stroke pathophysiology.

## Materials

All restriction enzymes were purchased from New England Biolabs (Milan, Italy) or Invitrogen (Milan, Italy). Luciferase reporter kits and vectors were from Promega (Milan, Italy). Synthetic oligonucleotides were from Primm (Milan, Italy). Dulbecco modified eagle medium (DMEM) and fetal bovine serum (FBS) were from Invitrogen. Small interference double-stranded RNA oligonucleotides (siRNA) against human Sp1 (GenBank accession number NM_181054) were from Dharmacon (Lafayette, CO, USA).

The siRNA against rat MTF-1 (Rn_LOC362591_1; Rn_LOC362591_2; Rn_LOC362591_3; Rn_LOC362591_4) and negative control siCONTROL (siCTL) (1027280) were from Qiagen (Milan, Italy). All common reagents were of the highest quality, purchased from Sigma-Aldrich (Milan, Italy).

### Cloning the ultrashort *Ncx1* promoter and site-directed mutagenesis

The short *Ncx1* promoter (−340/+151) has been previously realized^9^. The ultrashort *Ncx1* (−160/+151) promoter bearing 311 bp of the genomic sequence was obtained by PCR amplification of the short construct using a sense primer introducing a SacI site at position −160 (5’-TTTGAGCTCGAGCCAGTGCGAGGCTGCGGGCGG-3’) and an antisense primer from base +151 (5’-GATCTCGAGCCCGGGTCCTGAAAGC-3’). The amplified fragment was cloned in the pGL3basic vector using SacI and SmaI sites. Mutated constructs were generated using the QuickChange site-directed mutagenesis kit (Stratagene; Milan, Italy). Primers pairing to HRE1 at −164/−160 bp (sense: 5’-GCGGGCTGGGCTAAAGCGAGCCAGTGCG-3’ and antisense: 5’-CGCACTGGCTCGCTTTAGCCCAGCCCGC-3’) and pairing to HRE2 at −331/−327 bp (sense: 5’-GCGTGCTAGCCTTTCACTGCGGGGGC-3’ and antisense: 5’-GCCCCCGCAGTGAAAGGGCTAGCACGC-3’) were used to mutate the HREs from RCGTG to RAAAG. The plasmids carrying the single mutations were named short HRE1mut and short HRE2mut, accordingly to the HRE site mutated, respectively, and the last one containing both mutations was named short HRE1+2mut. Primers pairing to MRE1 at −23/−17 bp, (sense: 5’-CTCCCGCCCGCGAATTCCGCTGCCTGCTG-3’ and antisense: 5’-CAGCAGGCAGCGGAATTCGCGGGCGGGAG-3’), and pairing to the MRE2 at −88/−82 bp (sense: 5’-GAGCGAGGGAGGGAGAAAGCGCGCGCGCCGCCC-3’ and antisense: 5’-GGGCGGCGCGCGCGCATTTATCCCTCCCTCGCTC-3’) were used to mutate the MREs from GNGYGCA to GCGAATT. The vectors carrying the single mutations were named ultrashort MRE1mut and ultrashort MRE2mut, accordingly to the MRE site mutated, respectively, and the last one containing both mutations was named short MRE1+2mut.

### Transfection and reporter assay

Human neuroblastoma SH-SY5Y cells were grown as monolayers in DMEM supplemented with 10% heat-inactivated FBS, penicillin (100 U/mL), streptomycin (100 g/mL), non-essential amino acid (0.1 mmol/L), and L-glutamine (2 mmol/L). SH-SY5Y transfection was performed using a standard Ca^2+^ phosphate precipitation protocol^35^. In particular, 8 × 10^5^ cells were co-transfected with 2.4 μg of each plasmid containing the *Firefly* reporter gene and 0.6 μg of the *Renilla* luciferase plasmid. The precipitate was removed after 15 h. The luciferase activity was expressed as *Firefly*-to-*Renilla* ratio after a 24 h exposure to CoCl_2_ 100 μM. For RNA interference assay, a concentration of 100 nM of specific siCTL or siSp1, was used. Dual-luciferase assays were performed following the supplier’s instructions (Promega). The luciferase activities, recorded with a manual luminometer (Glomax 20/20, Promega), were measured in 20 µl of cell lysate.

### Western blotting

Cytoplasmic, nuclear, and total extracts were obtained as previously described^9,36^. Protein concentrations were determined by the Bio-Rad protein assay (Biorad; Milan, Italy). Specific antibodies were used: anti-MTF-1 (rabbit polyclonal, 1:500; Invitrogen); anti-NCX1 (rabbit polyclonal,1:1000; Swant), anti-Sp1 (rabbit polyclonal, 1:500; Santa Cruz Biotechnology; Milan, Italy); and anti β-actin (mouse monoclonal, 1:10000; Sigma Aldrich). Immunoreaction was revealed using antimouse and antirabbit immunoglobulin-G conjugated to peroxidase 1:2000 (GE-Healthcare; Milan, Italy) by the ECL reagent (GE-Healthcare). The optical density of the bands was determined by Chemi Doc Imaging System (Biorad) and normalized to the optical density of β-actin.

### Electrophoretic mobility shift assay

We used a non-radioactive procedure using fluorescence cyano-dye(Cy5)-labeled oligodeoxynucleotide duplexes as specific probes as previously reported^9,10^. Cy5 was tagged at the 5’ end oligonucleotides (MWG-Biotech AG; Eurofins; Ebersberg, Germany). The sequences of the sense strands of the duplexes used for electrophoretic mobility shift assay were as follows: NCX1-MRE1 wt:5’-CTCCCGCCCGCGCGCACCGCTGCC-3’; NCX1-MRE1-mut:5’-CTCCCGCCCGCGAATTCCGCTGCC-3’; hMTII-MREa:5’-AGCTTCGGGGCTTTTGCACTCGTCCCGGCTCTA-3’ and hMTII-MREa-mut:5’-AGCTTCGGGGCTTTGATGCTCGTCCCGGCTCTA-3’ as previously reported^37^.

The binding reaction performed in a volume of 20 µl with 5 µg of nuclear extracts, 400 ng of poly(dI-dC) (Roche), and 1 pmol of the Cy5-labeled probe in a buffer containing 10 mmol/L Hepes, pH 7.9, 100 mmol/L KCl, 0.2 mmol/L EDTA, 1 mmol/L MgCl2, 0.5 mmol/L DTT, and 10% glycerol. Competition studies were performed with varying concentrations of unlabeled competitor DNA. The reaction was performed on a 5% non-denaturing acrylamide gel in Tris borate-EDTA (30 mmol/L Tris, 30 mmol/L boric acid, 0.6 mmol/L EDTA). The gels were scanned at 600 V on a Typhoon 9400 imager using a green laser (633 nm) for excitation and a 670BP30 emission filter.

### In vivo experiments

#### Experimental groups

Male Sprague Dawley rats (Charles River; Milan, Italy) weighing 250–300 g were housed under diurnal lighting conditions (12 h darkness/light). Experiments were performed according to the international guidelines for care and use of experimental animals of the European Community Council directive (86/609/EEC). All experiments were approved by the Institutional Animal Care and Use Committee of “Federico II” University of Naples, Italy.

### Quantitative real-time PCR analysis

Rats were deeply anesthetized with 3% isoflurane vaporized in O_2_/N_2_O (50:50) and sacrificed. Cortices from mice were rapidly removed and immediately frozen on dry ice and stored at −80 °C until use. Total RNA was extracted with Trizol, following supplier’s instructions (Life Technologies; Monza, Italy) and cDNA was synthesized using 2 μg of total RNA with the High Capacity Transcription Kit following supplier’s instruction (Life Technologies) as previously reported^38^. qPCR was performed with TaqMan assays in a 7500 real-time PCR system (Life Technologies). Differences in mRNA levels were calculated as the difference in threshold cycle (2^−ΔΔCt^) between the target genes (*Slc8a1*TaqMan ID:Mm00441524_m1; MTF-1 ID:Mm00485724), and the reference gene: beta-glucuronidase (Gusb; ID:Mm00446953_m1).

### Transient Focal Ischemia and RLIP

Transient focal ischemia was induced as previously described^39^ by 100’ occlusion of the middle cerebral artery (MCA). In particular, a 5-O surgical monofilament nylon suture (Doccol, Sharon, MA) was inserted from the external carotid artery into the internal carotid artery and advanced into the circle of Willis up to the branching point of the MCA, thereby occluding the MCA. Achievement of ischemia was confirmed by monitoring regional cerebral blood flow in the area of the right MCA. Cerebral blood flow was monitored through a disposable microtip fiber optic probe (diameter 0.5 mm) connected through a Master Probe to a laser Doppler computerized main unit (PF5001; Perimed, Järfälla, Sweden) and analyzed using PSW Perisoft 2.519. Animals not showing a cerebral blood flow reduction of at least 70% were excluded from the experimental group, as well as animals that died after ischemia induction. Rectal temperature was maintained at 37 ± 0.5 °C with a thermostatically controlled heating pad and lamp. All surgical procedures were performed under an operating stereomicroscope.

Rats were randomly divided into 3 experimental groups: (i) sham-operated; (ii) ischemic; and (iii) remote post-conditioned rats. Sham-operated animals underwent the same experimental surgical procedure except that the filament was not introduced; in the ischemic group, the MCA was occluded for 100’. Remote limb ischemic postconditioning (RLIP) was induced by subjecting ischemic animals to a cycle of 20’ of femoral artery occlusion (FAO) as previously described^26^. Briefly, 20’ after MCA reperfusion, the femoral artery was identified, isolated, and occluded for 20’ using two microserrafine clips (FST). All animals were euthanized 24 h after the 20’ of FAO to quantify either the infarct volume or the protein and mRNA expression.

### Evaluation of the infarct volume

Animals were killed with sevoflurane 24 h after FAO or tMCAO. Brains were quickly removed, sectioned coronally at 1-mm intervals, and stained by immersion in the vital dye (2%) 2,3,5-triphenyltetrazoliumhydrochloride. The total infarct volume was calculated through image analysis software Image-Pro Plus^40^ by summing the infarction areas of each section and by multiplying the total by slice thickness (1 mm). Furthermore, to avoid that edema could affect the infarct volume value, infarct volume was expressed as a percentage of the ischemic damage by dividing the infarct volume calculated as above described by the total ipsilateral hemispheric volume^41^. In this way, any potential interference due to increased brain volume caused by water content increase is eliminated. The researcher who performed the image analysis was blinded to the study groups.

### siRNA administration

In rats positioned on a stereotaxic frame, a 23-gauge stainless-steel guide cannula (Small Parts) was implanted into the right lateral ventricle using the stereotaxic coordinates of 0.4 mm caudal to bregma, 2 mm lateral, and 2 mm below the dura^39^. The cannula was fixed to the skull using dental acrylic glue and small screws. The siRNA (5 μl, 5 μM) was administered intracerebroventricularly 3 times, 24 h and 18 h before and 6 h after MCA occlusion.

### Statistical analysis

The data were evaluated as means ± SEM. Statistically significant differences among means were determined by ANOVA followed by Bonferroni test. The number of animals included in each experimental group was pre-determined using G-power software; the threshold for statistical significance data was set at *p* < 0.05.

## Supplementary information

Legends supplemental figures

Figure S1

Figure S2
