# Correction: The hypoxia sensitive metal transcription factor MTF-1 activates NCX1 brain promoter and participates in remote postconditioning neuroprotection in stroke

**DOI:** 10.1038/s41419-023-05942-6

**Published:** 2023-07-31

**Authors:** Valeria Valsecchi, Giusy Laudati, Ornella Cuomo, Rossana Sirabella, Annalisa Del Prete, Lucio Annunziato, Giuseppe Pignataro

**Affiliations:** 1 Division of Pharmacology, Department of Neuroscience, Reproductive and Dentistry Sciences, School of Medicine, Federico II University of Naples, via Pansini 5, 80131 Naples, Italy; 2 IRCCS SDN, via Gianturco 113, 80143 Naples, Italy

**Keywords:** Cellular neuroscience, Preclinical research

Correction to: *Cell Death and Disease* 10.1038/s41419-021-03705-9, published online 30 April 2021

Following the publication of this article, the authors noted that Dr. Annalisa del Prete’s name was inadvertently omitted from the author list.

Annalisa Del Prete

Division of Pharmacology, Department of Neuroscience, Reproductive and Dentistry Sciences, School of Medicine, Federico II University of Naples, via Pansini 5, 80131 Naples, Italy

The original article has been updated.

