# Supplementary material for: The hypoxia sensitive metal transcription factor MTF-1 activates NCX1 brain promoter and participates in remote postconditioning neuroprotection in stroke
Source: Cell Death Dis. 2021 Apr 30;12(5):423. doi: 10.1038/s41419-021-03705-9 (PMC8087832; doi:10.1038/s41419-021-03705-9)
Supplement: Supplementary file 1 — Legends supplemental figures [file 41419_2021_3705_MOESM1_ESM.docx]

**LEGENDS OF SUPPLEMENTAL FIGURES**

**Figure S1. SP1 protein levels decreased in SH-SY5Y cells treated with a specific RNA interference for Sp1 and were not affected in the cortex of ischemic and postconditioned rats.** **A**, Representative Western blot of Sp1 in total extract from SH-SY5Y cells transfected with a non-targeting siRNA (siCTL) or with a siRNA for Sp1 (siSp1). *p<0.05 vs siCTL by t-test. Each column represents the mean±SEM of 6 independent experimental sessions, each one run in duplicate. **B**, Representative Western blot of Sp1 proteins in the cortex of rat: i) sham operated (CTL); ii) exposed to tMCAO (tMCAO); and iii) tMCAO+FAO. Each column represents the mean±SEM (n=5).

**Figure S2. MTF-1 protein levels decreased in the cortex of rat treated with a specific RNA interference for MTF-1.** Representative Western blot of MTF-1 in total extract from the cortex of rat icv-injected with a non-targeting siRNA (siCTL) or with a siRNA for MTF-1 (siMTF-1). *p<0.05 vs siCTL by t-test. Each column represents the mean±SEM (n=4).
