# Supplementary figures and images for: The hypoxia sensitive metal transcription factor MTF-1 activates NCX1 brain promoter and participates in remote postconditioning neuroprotection in stroke
Source: Cell Death Dis. 2021 Apr 30;12(5):423. doi: 10.1038/s41419-021-03705-9 (PMC8087832; doi:10.1038/s41419-021-03705-9)

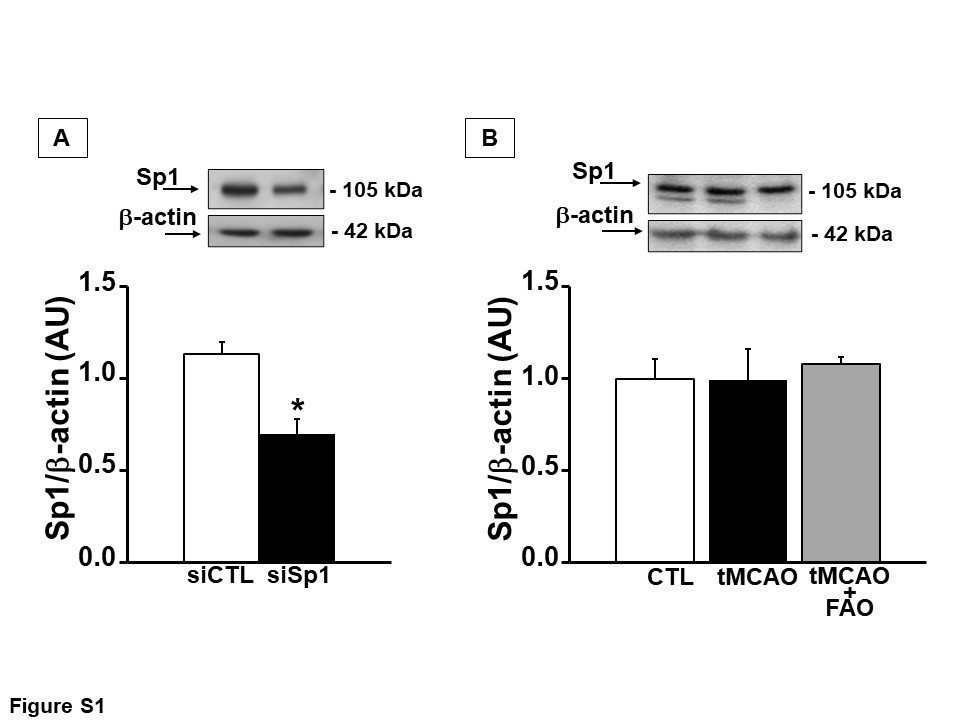

Supplement: Supplementary file 2 — Figure S1 [file 41419_2021_3705_MOESM2_ESM.jpg]

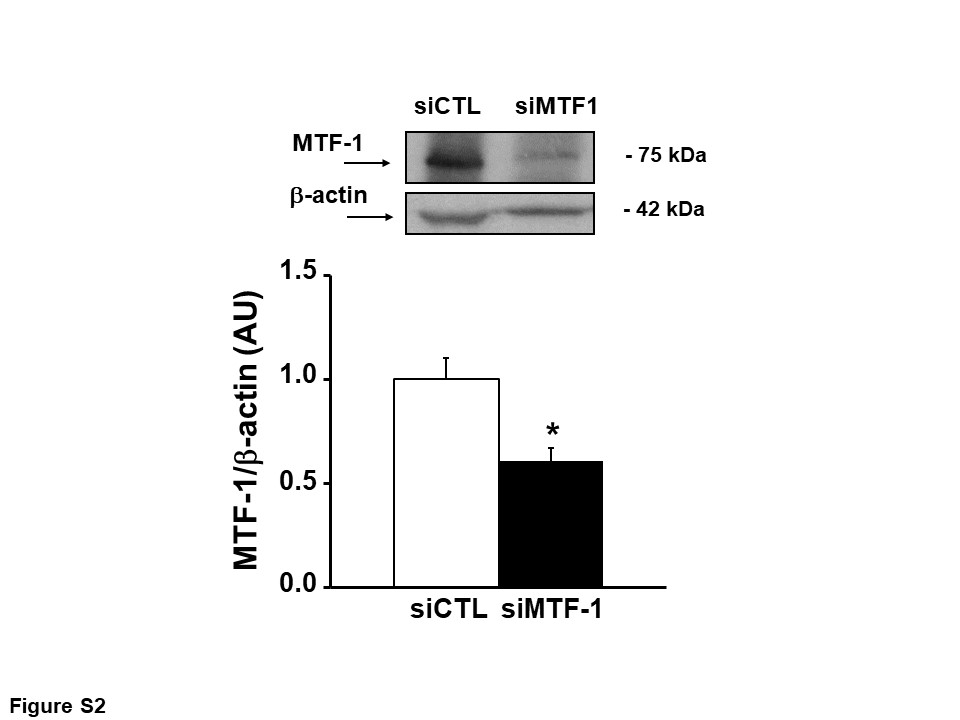

Supplement: Supplementary file 3 — Figure S2 [file 41419_2021_3705_MOESM3_ESM.jpg]
